# COVID-19 und Nierentransplantation

**DOI:** 10.1007/s11560-021-00485-3

**Published:** 2021-01-28

**Authors:** Florina Regele, Rainer Oberbauer

**Affiliations:** Univ. Klinik für Innere Medizin III, Klinische Abteilung für Nephrologie und Dialyse, Medizinische Universität Wien, Währinger Gürtel 18–20, 1090 Wien, Österreich

**Keywords:** Nierentransplantation, Coronavirus, Immunsuppression, Hyperinflammation, Antivirale Therapien, Kidney transplantation, Coronavirus, Immunosuppression, Hyperinflammation, Antiviral therapy

## Abstract

Nierentransplantierte Patienten stellen während der COVID-19(„coronavirus disease 2019“)-Pandemie eine spezielle Risikogruppe dar. Dies liegt sowohl an den häufig bestehenden Komorbiditäten als auch an der therapeutischen Immunsuppression. Letzterer kommt auch angesichts der stark zu Morbidität und Mortalität beitragenden Hyperinflammation eine komplexe Rolle zu. Bislang publizierte Fallserien zeigen eine hohe Hospitalisierungsrate und eine Mortalität zwischen 13 und 23 % in dieser Population. Die klinische Symptomatik sowie bislang etablierte Risikofaktoren scheinen jenen der Allgemeinbevölkerung zu ähneln. Eine heikle Frage in der Behandlung von an COVID-19 erkrankten Nierentransplantierten ist der Umgang mit der Immunsuppression, welche gemäß aktuellen Empfehlungen stufenweise und in Abhängigkeit vom klinischen Verlauf reduziert werden sollte. Auf der Suche nach wirksamen Therapien gegen SARS-CoV‑2 („severe acute respiratory syndrome coronavirus 2“) wurden zahlreiche in anderen Indikationen etablierte antivirale und antiinflammatorische Substanzen untersucht, wobei bislang nur für die Therapie mit Dexamethason bei Patienten mit Sauerstoffbedarf eine überzeugende Evidenz zu bestehen scheint. Zahllose Studien zu teils auch neuentwickelten Therapien laufen derzeit.

## Abstract (en)

The coronavirus disease 2019 (COVID-19) pandemic poses a particular risk for kidney transplant recipients. This is due to a high prevalence of comorbidities as well as therapeutic immunosuppression, which plays a complex role in view of the severe hyperinflammation contributing to morbidity and mortality. Many published case series including kidney transplant recipients reported a high proportion of hospitalized cases and mortality rates of 13–23%. The clinical symptoms and established risk factors for severe disease seem to be similar to those of the general population. The management of immunosuppressive treatment is a delicate question in the treatment of kidney transplant recipients with COVID-19. According to the current recommendations, a stepwise reduction should be carried out depending on the clinical course of the disease. Ongoing efforts to find an effective treatment for severe acute respiratory syndrome coronavirus type 2 (SARS-CoV-2) focus on repurposing known antiviral and anti-inflammatory substances. To date, only dexamethasone has shown to be an effective treatment in the subgroup of patients requiring oxygen supplementation; however, countless trials including novel therapeutic approaches are ongoing.

Seit dem Auftreten der ersten Fälle der durch SARS-CoV‑2 („severe acute respiratory syndrome coronavirus 2“) ausgelösten Erkrankung COVID-19 („coronavirus disease 2019“) im Dezember 2019 und seiner raschen Ausbreitung zu einer Pandemie mit weltweit über 60 Mio. Infizierten bis Ende November 2020 reißt das wissenschaftliche und öffentliche Interesse an der neuartigen Viruserkrankung nicht ab.

Zu den häufigsten Symptomen gehören respiratorische Symptome wie Husten oder Atemnot, Fieber, Muskelschmerzen, Kopfschmerzen, Verlust von Geruchs- und Geschmackssinn, aber häufig auch gastrointestinale Symptome wie Diarrhö [27]. Der Schweregrad der Erkrankung sowie der klinische Verlauf ist hierbei sehr variabel und reicht von asymptomatischen Infektionen über milde, grippeähnliche Verläufe bis hin zum schweren beatmungspflichtigen ARDS („acute respiratory distress syndrome“) mit hoher Mortalität [27].

Aufgrund der häufig milden oder asymptomatischen Verlaufsformen sowie des zeitweise eingeschränkten Zugangs zu Tests lag v. a. zu Beginn der Pandemie eine hohe Dunkelziffer an nicht diagnostizierten Fällen vor, wodurch die Mortalität der Erkrankung zu Beginn der Pandemie überschätzt wurde. Seroprävalenzstudien ergeben eine geschätzte globale Sterblichkeitsrate aller symptomatisch und asymptomatisch Infizierten („infection fatality rate“) von 0,15–0,20 % [25].

Der wichtigste Risikofaktor für einen schweren Verlauf ist ein höheres Lebensalter, wobei das Mortalitätsrisiko der Altersgruppe der über 80-Jährigen im Vergleich zur Altersgruppe der 50- bis 59-Jährigen um das 20-Fache erhöht ist [44]. Ebenso scheinen Männer und Patienten mit Diabetes, Adipositas, Asthma, chronischen Herz- und Lungenerkrankungen, Niereninsuffizienz und anderen Komorbiditäten ein höheres Mortalitätsrisiko aufzuweisen [13, 44].

## Schwere Verläufe durch Hyperinflammation

Bei einem geringen Anteil der an COVID-19 erkrankten Patienten kommt es zum Auftreten einer dysregulierten Immunantwort, welche meist in einer späteren Krankheitsphase auftritt. Im Rahmen dieser kommt es zu einer überschießenden Aktivierung des angeborenen Immunsystems mit erhöhter Freisetzung proinflammatorischer Zytokine und Makrophagenaktivierung bei gleichzeitig eingeschränkter T‑Zell-Antwort. Darüber hinaus dürfte es über die direkte Infektion von Endothelzellen über ACE2 („angiotensin-converting enzyme 2“), dem Rezeptor für das S‑Protein des SARS-CoV-2-Virus und damit dessen Eintrittspforte in die Zelle, sowie Aktivierung des Komplementsystems zu Endothelitis und prokoagulatorischen Veränderungen kommen, welche mit einem erhöhten Thrombose- und Embolierisiko von COVID-19 einhergehen [34]. Dies spiegelt sich auch im häufig deutlich erhöhten D‑Dimer bei kritisch kranken COVID-19-Patienten wider [20]. Letztlich führen all diese Veränderungen zu einem hyperinflammatorischen Zustand mit gestörter vaskulärer Barriere, Hyperkoagulopathie bis hin zum Multiorganversagen [10, 34, 35].

Rezente Studien stellen die Relevanz bzw. den Begriff des Zytokinsturms bei COVID-19 in Frage

Aufgrund der bei schweren COVID-19-Verläufen deutlich erhöhten proinflammatorischen Zytokine wie z. B. Interleukin(IL)-6, IL‑2 und Tumornekrosefaktor (TNF) sowie deren starker Assoziation mit Krankheitsschwere und Mortalität wurde die bei COVID-19 bestehende Hyperinflammation initial häufig mit dem Begriff des Zytokinsturms beschrieben, welcher von anderen Krankheitsentitäten wie ARDS oder in Assoziation mit der CAR(„chimeric antigen receptor“)-T-Zell-Therapie bekannt ist. Rezente Studien stellen allerdings die Relevanz bzw. den Begriff des Zytokinsturms bei COVID-19 in Frage, da die selbst bei schweren COVID-19-Fällen beschriebenen IL-6-Spiegel zwar deutlich erhöht sind, jedoch weit unter jenen liegen, die sonst bei Non-COVID-19-ARDS oder anderen mit einem Zytokinsturm einhergehenden Entitäten beschrieben werden. Die bei COVID-19 vorliegende systemische Inflammation dürfte sich somit von derjenigen bei anderen Krankheitsbildern wie Sepsis, ARDS oder dem durch CAR-T-Zellen induzierten „cytokine release syndrome“ unterscheiden, was wichtige therapeutische Implikationen mit sich bringt [28, 34, 40].

## COVID-19 bei Nierentransplantierten

### Inzidenz, Verlauf und Outcome

Patienten mit Nierenerkrankungen gehören zu einer speziellen Risikogruppe. Dies ist sowohl durch häufig bestehende Komorbiditäten wie Diabetes oder arterielle Hypertonie, aber auch durch die mit fortgeschrittener Niereninsuffizienz und Urämie einhergehende immunologische Dysfunktion bedingt. Darüber hinaus stellt die bei Patienten nach Nierentransplantation oder mit glomerulären Erkrankungen bestehende therapeutische Immunsuppression einen zusätzlichen Risikofaktor dar.

Das akute Nierenversagen stellt bei an COVID-19 erkrankten Nierentransplantierten eine häufige Komplikation dar

Ähnlich wie bei anderen Infektionserkrankungen scheint auch COVID-19 bei nierentransplantierten Patienten unter immunsuppressiver Therapie im Vergleich zur Normalbevölkerung häufiger einen schwereren Verlauf zu nehmen. Organtransplantierte haben altersunabhängig ein 6‑fach erhöhtes Risiko, an COVID-19 zu versterben [44].

Mittlerweile wurden zahlreiche Fallserien und Registerstudien zu COVID-19 bei Patienten nach Nierentransplantation publiziert (siehe Tab. 1). Die meisten davon beschreiben in erster Linie hospitalisierte und somit schwerer erkrankte Patienten, sodass sich über die Gesamtinzidenz und -mortalität in dieser Population nur eingeschränkte Aussagen treffen lassen. In einer französischen Studie fand man in einer Gesamtpopulation von 1216 aktiv kontaktierten Nierentransplantierten über 8 Wochen während der ersten Pandemiewelle eine Inzidenz von 5 % und eine Mortalität von 1 % [15]. Die meisten Studien zeigen im Vergleich zur Normalbevölkerung hohe Hospitalisierungsraten von 32–97 %. 7–32 % der Erkrankten müssen auf Intensivstationen behandelt werden. Die berichtete Mortalität unter allen Erkrankten lag je nach Studie bei 13–32 % (siehe Tab. 1).

**Table 1:** 

Autoren	Zentrum	Anzahl NTX-Patienten	Alter	Verlauf	Mortalität	Vorgehen hinsichtlich Immunsuppression	Risikofaktoren für Mortalität	Therapie
Akalin et al. [1]	Montefiore Medical Center, USA	36	60	78 % Hosp. 31 % intub. 17 % RRT	28 %	86 % Stopp Antimetabolit 21 % Stopp Tac	n. a.	86 % HCQ 21 % Leronlimab 7 % Toci 7 % Steroid
Lubetzky et al. [30]	Weill Cornell Medicine, USA	54	57	72 % hosp. 20 % ICU 39 % AKI 11 % TX-Verlust	13 %	44 % Stopp Antimetabolit 33 % Red. Tac	n. a.	62 % HCQ 4 % Rem 4 % Toc
Cravedi et al. (TANGO; [11])	Multizentrisch (Italien, Spanien, USA)	144	62	100 % Hosp.^a^ 29 % intub. 52 % AKI	32 %^a^	68 % Red./Stopp Antimetabolit 23 % Stopp Tac	Alter ↑, LDH ↑, IL‑6 ↑, PCT↑, Atemfrequenz↑	71 % HCQ 21 % Toci 14 % Virustatika
Fava et al. [17]	Multizentrisch (Spanien)	104	60	100 % Hosp.^a^ 55 % ARDS 13,5 % intub. 47 % AKI	27 %^a^	48 % Stopp CNI 12 % Stopp mTOR 68 % Stopp MMF	Alter ↑, LDH ↑, Lungenerkrankung	97 % HCQ 48 % LPV/r 34 % Toci 3 % DRV/r 5 % DRV/Cobi 2 % Rem 9 % IFβ1a
ERACODA [21]	Multizentrisch (26 Zentren in Europa)	305	60	89 % Hosp. 7 % ICU 4 % RRT	21 % (28d)	14 % red. CNI 15 % Stopp CNI 54 % Stopp MMF (% der Hosp.)	Alter, Prednisolon bei Aufnahme, Kreatinin ↑, Atemfrequenz ↑	73 % HCQ 18 % LPV/r 18 % Steroid 9 % Toci (% der Hosp.)
Caillard et al. [7]	Multizentrisch (Frankreich)	279	62	87 % Hosp. 32 % ICU 38 % AKI 10 % RRT 3 % TX-Verlust	15 % (23 % der Hosp.; 30 Tage)	28 % Stopp CNI 71 % Stopp Antimetabolit	Alter > 60, CVD, Dyspnoe	25 % HCQ 7,8 % Virustatika 5,3 % Toci
Mehta et al. [32]	Single Center, USA	44	59	77 % Hosp. 41 % AKI 30 % ICU	14 %	79 % Stopp Antimetabolit 18 % Red. Antimetabolit	n. a.	75 % HCQ
Crespo et al. [12]	Multizentrisch (Spanien)	414	62	92 % Hosp. 13 % ICU	26 % (44 Tage)	n. a.	Alter > 65, GI-Symptome protektiv	89 % HCQ 34 % LPV/r 18,6 % Toci 49 % Steroid
Bossini et al. [6]	Brescia, Italien	53	60	85 % Hosp. 19 % ICU 28 % AKI	28 %	79 % Stopp MMF+CNI 15 % Stopp MMF 17 % CNI halbiert	Alter, Dyspnoe bei Aufnahme, Tac (Trend)	79 % HCQ 60 % Virustatika
Elias et al. [15]	Paris, Frankreich	66	56	91 % Hosp. 22 % ICU 42 % AKI 11 % RRT	24 %	62 % Stopp MMF 4 % Stopp CNI 36 % unverändert	n. a.	11 % HCQ 2 % Toci 3 % Ecul
Demir et al. [14]	Multizentrisch (Türkei)	40	45	97 % Hosp. 17,5 % ICU	12,5 %	100 % Stopp Antimetabolit 63 % CNI halbiert	Abstoßungstherapie, CsA protektiv	100 % HCQ 45 % Favipiravir 12 % Toci
Husain et al. [24]	Columbia University Irving Medical Center, USA	41^b^ primär ambulante Pat	49	32 % Hosp.	0 %	63 % Red. IS	n. a.	n. a.
Benotmane et al. [5]	Straßburg, Frankreich	49	61	84 % Hosp. 28 % ICU 63 % AKI	18 % (30 Tage)	100 % Stopp MMF/mTOR 37 % Stopp CNI (% der Hosp.)	CRP ↑, IL‑6 ↑, D‑Dimer ↑, hsTropT ↑	37 % HCQ 65 % AZT 12 % LPV/r 47 % Steroid 10 % Toci (% der Hosp.)

*NTX *Nierentransplantation,* AKI* akutes Nierenversagen („acute kidney injury“), *RRT* Dialysepflichtigkeit („renal repplacement therapy“), *TX* Transplantat, *Hosp.* Hospitalisationen, *intub.* intubiert, *Red.* Reduzierung, *ICU* Intensive Care Unit, *MMF* Mycophenolat-Mofetil, *ARDS* „acute respiratory distress syndrome“, *IF* Interferon, *IL* Interleukin, *CVD* Herz-Kreislauf-Erkrankung („cardiovascular disease“), *GI* gastrointestinal, *LDH* Laktatdehydrogenase, *PCT* Procalcitonin, *CsA* Ciclosporin A, *mTOR* „mechanistic target of rapamycin“, *CNI* Calcineurininhibitoren, *IS* Immunsuppression, *TAC* Tacrolimus, *HCQ* Hydroxchloroquin, *Toci* Tocilizumab, *LPV/r* Lopinavir/Ritonavir, *Rem* Remdesivir, *AZT* Azithromycin, *DRV/r* Darunavir/Ritonavir, *DRV/Cobi* Darunavir/Cobicistat, *CRP* C-reaktives Protein, *hsTropT* hoch sensitives Troponin T

^a^nur hospitaliserte Patienten inkludiert

^b^suspizierte und bestätigte Fälle

Das initiale klinische Bild ähnelt dem der Allgemeinbevölkerung mit Fieber und Husten als häufigste Symptome. Diarrhö scheint mit 20–50 % vergleichsweise häufiger aufzutreten [1, 7, 11, 12, 17, 21, 30, 32]. Das akute Nierenversagen („acute kidney injury“, AKI) stellt bei an COVID-19 erkrankten Nierentransplantierten eine häufige Komplikation dar. In den größeren Fallserien lag die Inzidenz bei 28–63 % aller Erkrankten, bei 4–17 % war eine vorübergehende Nierenersatztherapie notwendig [1, 6, 7, 11, 15, 17, 21, 30, 32]. Auch in der Allgemeinbevölkerung tritt das AKI als Komplikation von COVID-19 mit einer Inzidenz von bis zu 36 % der Hospitalisierten deutlich häufiger auf, als anfänglich berichtet [9, 22, 33]. Die Genese des AKI bei COVID-19 ist vermutlich multifaktoriell bedingt. Häufig tritt das AKI im Rahmen eines Multiorganversagens mit Hyperinflammation auf. Mehrere Studien weisen jedoch auch auf die Nieren als direktes Ziel von SARS-CoV‑2 hin. So konnte immunhistochemisch eine Akkumulation von SARS-CoV‑2 in den Nierentubuli nachgewiesen werden [16, 42]. Letztlich ist es noch unklar, inwieweit das häufig beobachtete AKI sowie andere berichtete renale Pathologien wie Proteinurie oder Hämaturie durch direkte zytopathische Schädigung durch das Virus selbst verursacht werden oder ob die Mehrheit der Fälle als eine Mitbeteiligung im Rahmen eines systemischen Multiorganversagens, renaler Minderperfusion und Hyperinflammation zu sehen ist.

### Risikofaktoren für schwere Verläufe

In mehreren der oben zitierten Studien wurden Risikofaktoren für schwere Verläufe und Mortalität von COVID-19 bei Nierentransplantierten analysiert. Ähnlich wie bei der Allgemeinbevölkerung fanden fast alle Studien ein höheres Alter als Hauptrisikofaktor für Mortalität [4, 6, 11, 12, 17, 21]. Eine spanische Studie mit 104 mit COVID-19 hospitalisierten Patienten zeigte, dass das Alter zwar einen Risikofaktor für Mortalität, nicht jedoch für die Entwicklung eines ARDS darstellte [17]. Weitere beschriebene Risikofaktoren für Mortalität waren Dyspnoe/Tachypnoe bei Aufnahme, kardiovaskuläre oder pulmonale Vorerkrankungen sowie mehrere Biomarker, wie IL‑6, C‑reaktives Protein (CRP), Procalcitonin (PCT), D‑Dimer und hoch sensitives Troponin T (hsTropT), deren Erhöhung als Ausdruck eines hyperinflammatorischen Zustands als Folge der SARS-CoV-2-Infektion mit besonders schweren Verläufen assoziiert ist [4, 6, 11, 12, 17, 21]. In einer großen spanischen Registerstudie war das Auftreten von gastrointestinalen Symptomen allein oder in Kombination mit respiratorischen Symptomen mit einem günstigeren Krankheitsverlauf assoziiert [12].

Angesichts der erhöhten Infektanfälligkeit durch die therapeutische Immunsuppression auf der einen Seite und der potenziell abschwächenden Effekte auf die zu Krankheitsschwere und Mortalität beitragende Hyperinflammation auf der anderen Seite stellt sich die Frage, inwieweit sich die einzelnen immunsuppressiven Substanzen auf den Krankheitsverlauf auswirken.

Dieser Zusammenhang wurde in mehreren Fallserien untersucht. Die meisten davon fanden keinen Einfluss der Erhaltungsimmunsuppression auf Mortalität oder Hospitalisierungsrate [6, 7, 11, 12, 17]. Eine Analyse der European Renal Association COVID-19 Database (ERACODA), welche 305 an COVID-19 erkrankte Nierentransplantierte inkludierte, zeigte eine höhere Mortalität jener Patienten, die bei Aufnahme unter Therapie mit Aprednisolon standen (Hazard Ratio [HR]: 2,88: 95 %-Konfidenzintervall [KI]: 1,03–8,03; [21]).

Eine französische Studie mit 49 Nierentransplantierten ergab höhere Hospitalisierungsraten jener Patienten, die mit Mycophenolat-Mofetil (MMF) im Vergleich zu mTOR(„mechanistic target of rapamycin“)-Inhibitoren behandelt wurden [5]. In einer türkischen Studie fand sich ein protektiver Effekt von Cyclosporin A (CsA) hinsichtlich der Mortalität bei 40 Nierentransplantierten [14]. Weder die Zeit nach Transplantation noch die Induktionstherapie scheinen einen Einfluss auf die Mortalität oder die Schwere des Verlaufs zu haben [6, 7, 11, 12, 17].

## Viruselimination und immunologische Response bei Nierentransplantierten

Über die Dynamik der Viruselimination und die Dauer des positiven Virusnachweises bei transplantierten Patienten gibt es nur wenig Daten, auch wenn einzelne Fallberichte von prolongiert positiven Nasenrachenabstrichen bis zu 57 Tage nach Erkrankung berichten [19, 45]. Der initiale C_T_(„cycle threshold“)-Wert, welcher bei der Real-time-PCR („polymerase chain reaction“) die Viruslast anzeigt, scheint zwar bei aus dem Plasma, nicht jedoch bei aus dem Nasenrachenabstrich gewonnenen Proben mit der Krankheitsschwere und der Mortalität zu korrelieren [4].

Eine Studie verglich den immunologischen Phänotyp von 18 nierentransplantierten und an COVID-19 erkrankten Patienten mit dem von 36 gematchten nichtinfizierten Nierentransplantierten [4]. Hierbei zeigte sich, dass es auch unter Immunsuppression zu einer adäquaten adaptiven Immunantwort kam, die mit jener der Allgemeinbevölkerung vergleichbar war. Analog zur nichttransplantierten Population fanden sich bei den infizierten Nierentransplantierten niedrigere Lymphozytenzahlen mit weniger absoluten T‑Zellen und innerhalb der T‑Zell-Population eine erhöhte CD4+/CD8+-Ratio. Der in anderen Studien beschriebene erhöhte Anteil an dysfunktionalen T‑Zellen wurde in dieser Studie nicht beobachtet. Parameter, welche mit einem schwereren Krankheitsverlauf einhergingen, waren Lymphopenie, eine erhöhte Ratio von Neutrophilen zu Lymphozyten und erhöhte IL-6-Spiegel. Die Krankheitsschwere hatte keinen Einfluss auf die T‑ und B‑Zell-Subsets. Im Unterschied zur COVID-19-positiven Allgemeinbevölkerung fand sich bei infizierten Nierentransplantierten eine geringere Anzahl an regulatorischen T‑Zellen. Die meisten Patienten entwickelten Anti-SARS-CoV-2-IgM (Immunglobulin M) und IgG-Antikörper ab dem 10. Tag nach Symptombeginn, wobei Pausieren oder Weiterführung der Therapie mit MMF/MPA keinen Einfluss auf die Antikörperentwicklung hatte. Ebenso wenig hatte die Krankheitsschwere einen Einfluss auf die Antikörperbildung [4].

## Management der Immunsuppression

Eine zentrale Frage in der Behandlung von Nierentransplantierten, welche an COVID-19 erkranken, ist jene nach dem Umgang mit der bestehenden Immunsuppression. Hierbei gilt es, das Risiko eines schweren Krankheitsverlaufs durch eine beeinträchtigte Virusbekämpfung gegen das Risiko einer Abstoßung sowie die Gefahr einer aggravierten Hyperinflammation nach Reduktion oder Pausieren der immunsuppressiven Therapie abzuwägen.

In den meisten publizierten Fallserien wurde die Therapie mit Antimetaboliten bei einer Mehrheit der Patienten abgesetzt oder reduziert und je nach Krankheitsschwere auch die Calcineurininhibitoren (CNI) häufig reduziert. (siehe Tab. 1) Hierbei ist anzumerken, dass im Großteil dieser Studien hospitalisierte und schwerer erkrankte Patienten tendenziell überrepräsentiert sind. Ausgehend von den bisher publizierten Erfahrungen größerer Transplantzentren und Fachgesellschaften, wurden von der ERA(European Renal Association)-EDTA(European Dialysis and Transplant Association)-DESCARTES-Arbeitsgruppe Empfehlungen zur Reduktion der Immunsuppression bei Nierentransplantierten mit COVID-19 herausgegeben [31].

Diese empfiehlt eine stufenweise Reduktion der Immunsuppression, wobei bei mild symptomatischen Patienten zunächst MPA (Mycophenolsäure)/Azathioprin/mTOR-Inhibitor pausiert oder auf Low-dose-Steroide umgestellt werden sollte. Bei Patienten mit Hinweis auf das Vorliegen einer Pneumonie sollte je nach bestehenden Risikofaktoren die Immunsuppression entweder auf 15–20 mg/Tag Aprednisolon umgestellt oder eine duale Immunsuppression, bestehend aus niedrig dosiertem CNI und Steroiden angestrebt werden. Bei schwer erkrankten Patienten sollte die bestehende Immunsuppression komplett durch 15–25 mg Aprednisolon ersetzt werden.

## Therapeutische Ansätze

Seit dem Beginn der Pandemie wird weltweit mit Hochdruck an der Entdeckung von therapeutischen Möglichkeiten gegen COVID-19 gearbeitet. Neben der Entwicklung von Impfstoffen werden verschiedenste antivirale und immunmodulatorische Substanzen auf ihre Wirksamkeit gegen COVID-19 untersucht. Bei der Behandlung von Nierentransplantierten muss hierbei auch ein Augenmerk auf potenzielle nephrotoxische Nebenwirkungen sowie die Interaktion mit laufender immunsuppressiver Medikation gelegt werden.

### Antivirale Substanzen

#### Remdesivir

Das Nukleosidanalogon Remdesivir wurde ursprünglich für die Behandlung von Ebola entwickelt. Ausgehend von seiner In-vitro-Wirksamkeit gegen zahlreiche andere humanpathogene RNA-Viren wie SARS-CoV‑1 und MERS(„Middle East respiratory syndrome“)-CoV‑2 wurde es bereits früh in der Pandemie gegen SARS-CoV-2-Infektionen eingesetzt und war das erste Therapeutikum, welches durch die US-amerikanische Food and Drug Administration (FDA) zur Therapie zugelassen wurde. In einer multizentrischen, randomisierten Studie mit 1063 Patienten zeigte sich unter Remdesivir eine im Vergleich zu Placebo kürzere Zeit bis zur Genesung (10 vs. 15 Tage; „ratio for recovery“: 1,29; 95 %-KI: 1,12–1,29), jedoch ohne Einfluss auf die 28-Tages-Mortalität. Der Nutzen war bei den früh nach Symptombeginn behandelten Patienten am größten. Gemäß den neuesten Daten aus dem WHO (World Health Organization) Solidarity Trial konnte jedoch bei etwa 5000 randomisierten Patienten im Vergleich zu Placebo kein Effekt auf Hospitalisierungsdauer, Beatmungsrate oder Mortalität nachgewiesen werden [3, 43].

#### Lopinavir/Ritonavir

Das HIV-Medikament Lopinavir/Ritonavir wurde aufgrund seiner *in vitro* hemmenden Wirkung auf SARS-CoV‑1 bei COVID-19 eingesetzt. Eine randomisierte Studie mit 199 Patienten konnte jedoch keinen Nutzen im Vergleich zu SOC („standard of care“) zeigen [8]. Ebensowenig erbrachte der Solidarity Trial den Nachweis einer verringerten Hospitalisierungsdauer, Beatmungsrate oder Mortalität im Vergleich zu Placebo [43].

Beim Einsatz bei Nierentransplantierten sollte der durch Ritonavir gehemmte Tacrolimusmetabolismus mit konsekutiv stark erhöhten Tacrolimusspiegeln beachtet und eine Therapie mit CNI nach Möglichkeit stark reduziert oder pausiert werden.

#### Hydroxychloroquin

Das Malariamedikament Hydroxychloroquin (HCQ) wurde, teils in Kombination mit Azithromycin, aufgrund seiner *in vitro* nachgewiesenen antiviralen Wirksamkeit verbreitet in der Behandlung von COVID-19 eingesetzt. Die von der FDA im März 2020 erteilte Notfallzulassung wurde nach zahlreichen negativen Studien sowie teils auch Sicherheitsbedenken im Juni 2020 wieder zurückgezogen.

Beim Einsatz von Lopinavir/Ritonavir bei Nierentransplantierten ist der gehemmte Tacrolimusmetabolismus zu beachten

Auch die größten, rezenten Studien RECOVERY und die interimistischen Ergebnisse des WHO Solidarity Trial konnten keinen Nutzen von HCQ in der Behandlung von COVID-19 nachweisen [36, 43].

### Antiinflammatorische Therapie

#### Steroide

Der Einsatz von hoch dosierten Steroiden in der Behandlung von COVID-19 war zu Beginn der Pandemie umstritten. In frühen Studien an mit SARS-CoV‑2, SARS-CoV‑1 oder MERS-CoV infizierten Patienten wurde aufgrund von fraglicher Wirksamkeit und potenziell negativen Effekten wie verzögerter Viruselimination, längerer Hospitalisierungsdauer und schädlichen Langzeiteffekten vor einem allzu liberalen Einsatz außerhalb von Studien gewarnt [29, 38].

Mittlerweile liegen jedoch auch vorläufige Ergebnisse der randomisierten RECOVERY-Studie vor, in welcher 2104 mit 6 mg Dexamethason behandelte Patienten mit 4321 Patienten in der SOC-Gruppe verglichen wurden. Die 28-Tages-Mortalität in der Dexamethasongruppe war v. a. bei den zum Randomisierunsgzeitpunkt invasiv beatmeten Patienten deutlich niedriger als in der SOC-Gruppe (29,3 % vs. 41,4 %; Rate Ratio [RR]: 0,64; 95 %-KI: 0,51–0,81). Bei jener Patientengruppe, die zum Zeitpunkt der Randomisierung keinerlei respiratorischen Support benötigte, zeigte sich jedoch kein Unterschied in der Mortalität. Darüber hinaus war die Risikoreduktion auch nur bei Patienten mit einer bei Einschluss bestehenden Krankheitsdauer von mehr als 7 Tagen nachweisbar [37]. Auch eine Metaanalyse von 7 randomisierten, kontrollierten Studien zeigte eine reduzierte 28-Tages-Mortalität unter mit Steroiden behandelten kritisch kranken Patienten [41]. In einer Beobachtungsstudie fand sich bei Patienten mit einem CRP von mehr als 20 mg/dl ein reduziertes Mortalitätsrisiko durch Behandlung mit Glukokortikoiden, wohingegen bei Patienten mit einem CRP von weniger als 10 mg/dl und Glukokortikoidtherapie ein erhöhtes Risiko für Mortalität und mechanische Beatmung bestand [26]. Zusammenfassend scheint somit ein Nutzen einer Steroidtherapie nur bei kritisch kranken Patienten mit Sauerstoffbedarf und längerer Krankheitsdauer zu bestehen.

#### Tocilizumab, Anakinra, Sarilumab

Basierend auf der Beobachtung, dass ein Teil der kritisch kranken COVID-19-Patienten einen hyperinflammatorischen Zustand mit deutlich erhöhten IL-6-Spiegeln entwickelt, wurden unterschiedliche monoklonale Antikörper gegen IL, allen voran der IL-6-Rezeptor-Antagonist Tocilizumab, in der Behandlung von COVID-19 eingesetzt.

Vorläufige Daten einer randomisierten, doppelblinden, Phase-III-Studie zeigen jedoch weder hinsichtlich der Mortalität noch hinsichtlich einer rascheren klinischen Besserung einen Nutzen durch Tocilizumab [18]. Auch für den IL-6-Rezeptor-Antagonisten Sarilumab konnte in einer Phase-III-Studie keine verbesserte Mortalität bei beatmeten Patienten nachgewiesen werden [46].

Aufgrund der Ähnlichkeit des hyperinflammatorischen Stadiums bei COVID-19 mit Formen des sekundären Hämophagozytosesyndroms wurde auch der IL-1-Antagonist Anakinra in der Behandlung von COVID-19 eingesetzt. In einer Studie mit 52 kritisch kranken COVID-19-Patienten konnten durch die Behandlung mit Anakinra eine im Vergleich zu einer historischen Kontrollgruppe verbesserte Mortalität und eine geringere Beatmungspflichtigkeit erzielt werden [23].

### Antivirale Antikörpertherapien

#### Rekonvaleszentenplasma

Einen anderen Ansatz verfolgt die Behandlung mit Rekonvaleszentenplasma, durch welches neutralisierende Antikörper transfundiert werden. Eine rezente randomisierte Studie an 333 Patienten mit schwerem COVID-19-Verlauf konnte jedoch nach 30 Tagen keine Unterschiede hinsichtlich des klinischen Zustands oder der Mortalität im Vergleich zu Placebo feststellen [39].

#### Antiviraler Antikörpercocktail (Casivirimab und Imdevimab)

Eine Therapie, die durch ihre Anwendung bei der Erkrankung des amerikanischen Präsidenten Donald Trump die mediale Aufmerksamkeit auf sich zog, ist ein antiviraler Antikörpercocktail. Dieser enthält die beiden monoklonalen Antikörper Casivirimab und Imdevimab, welche sich gegen unterschiedliche, nicht überlappende Domänen des viralen Spike-Proteins richten. Der Vorteil der Verwendung eines Antikörperpaars liegt darin, dass der virale „Escape-Mechanismus“ durch Bildung von resistenten Mutanten verhindert wird, da in diesem Fall der zweite Antikörper das Virus neutralisiert. Diese Therapie wird derzeit in mehreren Phase-II- und Phase-III-Studien untersucht [2].

## Fazit für die Praxis

- An COVID-19 („coronavirus disease 2019“) erkrankte nierentransplantierte Patienten weisen eine hohe Hospitalisierungsrate und Mortalität auf.
- Die klinische Symptomatik ähnelt jener der Allgemeinbevölkerung, wobei gastrointestinale Symptome vergleichsweise häufig aufzutreten scheinen.
- Die immunsuppressive Therapie sollte nicht präventiv reduziert werden. Im Erkrankungsfall wird je nach Krankheitsschwere eine duale Therapie mit Calcineurininhibitor (CNI) und Steroid empfohlen, bei schwerem Verlauf eine Überbrückung mit 15–25 mg Aprednisolon als Monotherapie.
- Unter den zahlreichen bei COVID-19 untersuchten medikamentösen Therapien konnte bislang nur für Dexamethason bei sauerstoffpflichtigen Verläufen eine Mortalitätsreduktion gezeigt werden.
